# Predicting material properties by integrating high-throughput experiments, high-throughput ab-initio calculations, and machine learning

**DOI:** 10.1080/14686996.2019.1707111

**Published:** 2020-01-15

**Authors:** Yuma Iwasaki, Masahiko Ishida, Masayuki Shirane

**Affiliations:** a Central Research Laboratories, NEC Corporation, Tsukuba, Japan; b PRESTO, Japan Science and Technology Agency, Saitama, Japan; c Nanoelectronics Research Institute (NeRI), National Institute of Advanced Industrial Science and Technology (AIST), Tsukuba, Japan

**Keywords:** Materials informatics, machine learning, combinatorial, high-throughput, ab-initio, 404 Materials informatics / Genomics

## Abstract

High-throughput experiments (HTEs) have been powerful tools to obtain many materials data. However, HTEs often require expensive equipment. Although high-throughput ab-initio calculation (HTC) has the potential to make materials big data easier to collect, HTC does not represent the actual materials data obtained by HTEs in many cases. Here we propose using a combination of simple HTEs, HTC, and machine learning to predict material properties. We demonstrate that our method enables accurate and rapid prediction of the Kerr rotation mapping of an Fe_x_Co_y_Ni_1-x-y_ composition spread alloy. Our method has the potential to quickly predict the properties of many materials without a difficult and expensive HTE and thereby accelerate materials development.

## GRAPHICAL ABSTRACT

**Figure:**
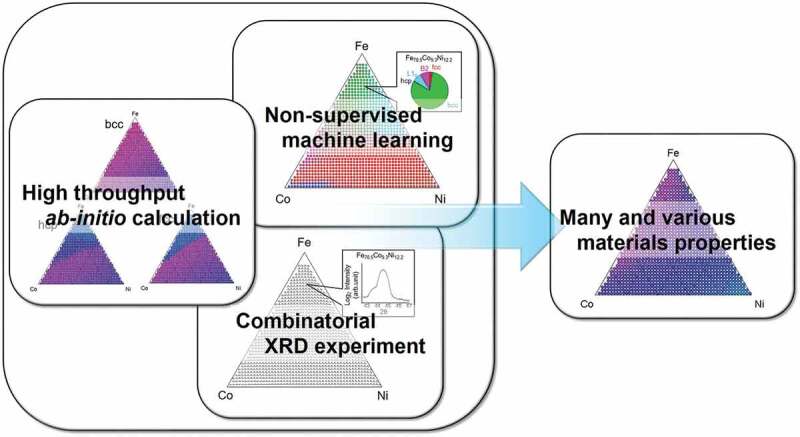

## 1. Introduction

Recent progress in high-throughput experiments (HTEs) has enabled rapid mapping of composition-structure-property relationships across a large compositional phase space [1–4] and has helped accelerate materials development. For example, Takeuchi et al. discovered new ferromagnetic materials using only two processes, combinatorial sputtering and scanning SQUID (superconducting quantum interference device) microscopy [5]. However, HTEs often require expensive equipment.

High-throughput ab-initio calculation (HTC) has also helped accelerate materials development [6]. Nishijima et al., for example, performed HTC on the basis of density function theory and identified better materials for lithium-ion battery cathodes [7]. HTC has the potential to perform composition-property mapping faster than HTE.

**Figure 1.**
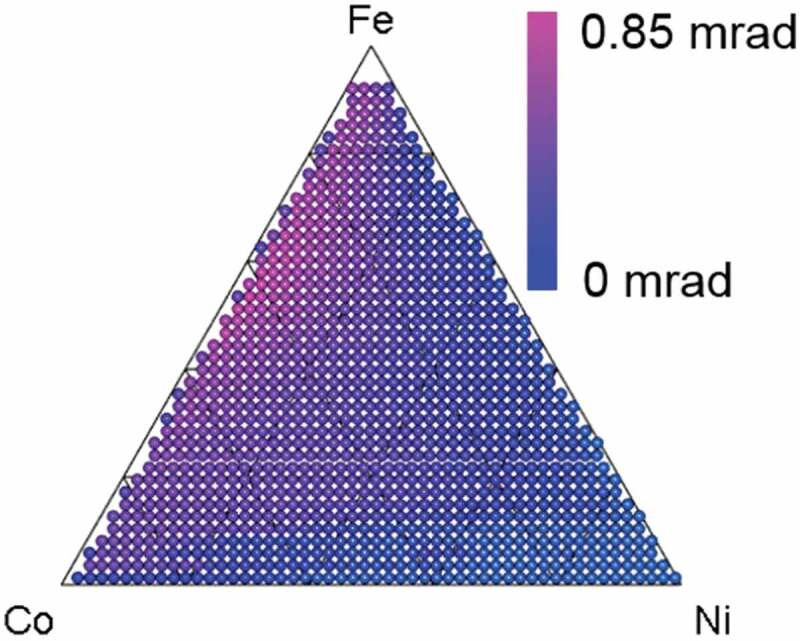
Mapping of Kerr rotation *θ_K_* of Fe_x_Co_y_Ni_1-x-y_ composition spread alloy from SMOKE experiment. Figure produced using data from Yoo et al. [8]. Kerr rotation is proportional to magnetic moment and/or saturation magnetization

**Figure 2.**
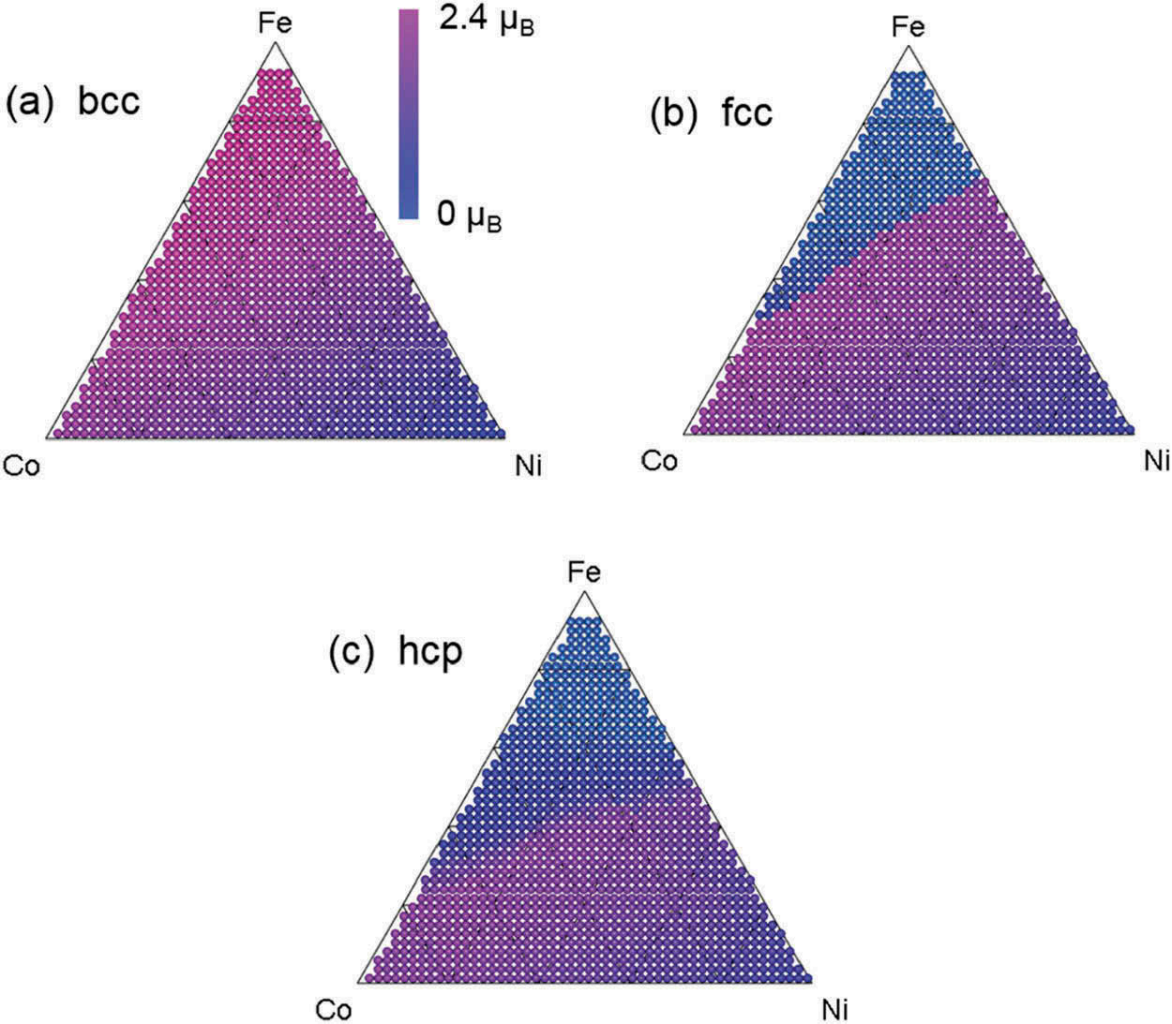
Predicted mapping of magnetic moment of Fe_x_Co_y_Ni_1-x-y_ composition spread alloy by Korringa Kohn Rostoker–Coherent Potential Approximation (KKR-CPA) ab-initio calculation. (a), (b), and (c) show results for bcc, fcc, and hcp structural phases, respectively. These results predicted by ab-initio calculation alone cannot reproduce experimental results shown in Figure 1

However, in many cases, HTC does not represent the actual composition-property map due to differences in the experimental data conditions (for HTE) and calculation data conditions (for HTC). Yoo et al., for example, presented an experimental mapping of Kerr rotation *θ_K_* for an Fe_x_Co_y_Ni_1-x-y_ composition spread alloy [8]. It was obtained from a combinatorial surface magneto-optic Kerr effect (SMOKE) experiment, which requires large and expensive equipment. The Kerr rotation was roughly proportional to saturation magnetization *M*, $\left|\theta_{k}\right|=K_{s}M$, where *K_s_* is a coefficient between 0.2 and 2.0 deg/T [9]. Since saturation magnetization *M* is almost proportional to magnetic moment *m* [10], *m* is a critical descriptor of *θ_K._* Figure 2(a–c) show the predicted mapping of the magnetic moment for the Fe_x_Co_y_Ni_1-x-y_ composition spread alloy for each structural phase (bcc, fcc, and hcp) obtained by ab-initio calculation. We used the Korringa-Kohn-Rostoker (KKR) Green function method, which enables the disordered phases to be calculated by coherent potential approximation (CPA) [11]. We used the Akai-KKR package for the KKR-CPA ab-initio calculation [12]. It is clear that the experimental mapping (Figure 1) cannot be predicted by ab-initio calculation alone because of the difference in structural phases. The ab-initio calculation works only for a single structural phase while actual materials used in HTEs often include a mixture of various structural phases.

Reducing the structural difference between HTE and HTC would enable predicting various properties more rapidly and accurately on the basis of HTC and thereby lead to further acceleration in materials development. We propose combining a simple HTE with HTC and machine learning (ML) for property prediction. ML methods enable rapid analysis of materials big data and have proven to be effective in the development of various materials including potential magnets [13], ferroelectrics [14], superconductors [15] and thermoelectrics [16,17]. We demonstrate that an experimental mapping of Kerr rotation *θ_K_* on a composition spread Fe_x_Co_y_Ni_1-x-y_ alloy can be predicted with our proposed method instead of with a difficult and expensive HTE (e.g., a combinatorial SMOKE experiment).

## 2. Proposed method

Our proposed property mapping method comprises four steps. As an example application, we describe the steps for predicting the experimental mapping shown in Figure 1 for Kerr rotation *θ_K_* for the Fe_x_Co_y_Ni_1-x-y_ composition spread alloy synthesized by Yoo et al. [8]. An Fe_x_Co_y_Ni_1-x-y_ composition spread thin film (100 nm) was fabricated on a sapphire (0001) substrate by combinatorial ion-beam sputtering deposition, followed by post-annealing in vacuum at 600 °C and 10^−8^ Torr for 3 hours. The details are explained elsewhere [8].

**Figure 3.**
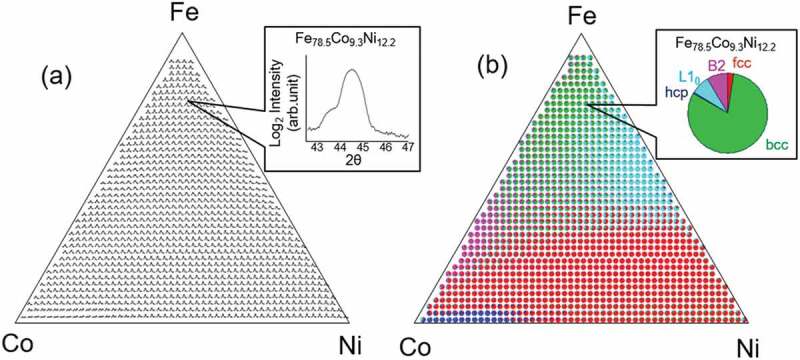
Structural phase diagram of Fe_x_Co_y_Ni_1-x-y_ composition spread alloy. (a) Mapping of XRD curves obtained from combinatorial XRD experiment. Inset shows XRD curve of Fe_78.5_Co_9.3_Ni_12.2_ composition. (b) Mapping of structure rate R derived by applying NMF to XRD curves shown in Figure 3(a). Inset shows pie chart for the structure rate of Fe_78.5_Co_9.3_Ni_12.2_ composition

First, a combinatorial X-ray diffraction (XRD) experiment, a common and simple type of HTE, was performed for the composition spread sample to obtain comprehensive XRD curves [8]. Figure 3(a) shows the many XRD data points for the composition spread Fe_x_Co_y_Ni_1-x-y_ alloy. The inset shows the XRD curve for Fe_78.5_Co_9.3_Ni_12.2_. The XRD data were obtained using a scanning microbeam X-ray diffractometer with a spatial resolution of 50–300 μm. The details are explained elsewhere [8].

Next, the structure rate was extracted from each XRD curve, which is not so easy. In the Fe-rich region, the structure was bcc. Similarly, the structures were fcc and hcp in the Ni- and Co-rich regions, respectively. However, an Fe_78.5_Co_9.3_Ni_12.2_ composition, for example, may have a mixture of bcc–fcc–hcp structural phases. Moreover, it may include ordered phases such as B_2_ and L_10_ phases. If this is the case, the XRD data can be curve–fitted and decomposed into single structural XRD curves to obtain the structure rate information. However, with combinatorial XRD, it takes a long time for curve-fitting many XRD data points one by one.

Therefore, we used non-supervised ML to decompose the XRD data into single structural XRD curves. Kusne et al. demonstrated that non-negative matrix factorization (NMF) enables combinatorial XRD curves to be quickly decomposed into single structural XRD curves, so the structure rate can be obtained immediately [13,18]. We performed NMF on an *R* programming language, where an ‘NMF’ package was used with the ‘Brunet’ method [19].

Figure 3(b) shows a structure rate mapping. Pie charts showing structure rate *R_structure_* (*R_bcc_, R_fcc_, R_hcp_, R_B2_, and R_L10_*) are plotted as a function of composition. The inset in Figure 3(b) shows an example pie chart for Fe_78.5_Co_9.3_Ni_12.2_. It shows a large number of bcc disordered phases and a small number of B2 and L1_0_ ordered phases. These results agree with previous results [20].

HTC was then performed to obtain magnetic moment *m* for each composition and structural phase. In addition to the magnetic moment mapping for the disordered phases (*m_bcc_, m_fcc_*, and *m_hcp_*) shown in Figure 2(a–c), the *m_B2_* and *m_L10_* for FeCo-B2 and FeNi-L1_0_ ordered phases were calculated.

Next, the weighted sum of structure rate *R_structure_* and magnetic moment *m_structure_* were calculated for each structural phase to reduce the structural difference between the experimental data (HTE, Kerr rotation *θ_K_*) and the calculation data (HTC, magnetic moment *m*). The value of the weighted sum should be proportional to Kerr rotation *θ_K_*.

(1) $$\theta_{K}\approx \overset{\;}{\underset{structure}{\sum }}R_{structure}\cdot m_{structure}$$

By following these steps, we can predict the mapping of Kerr rotation *θ_K_* on the basis of a simple HTE (combinatorial XRD), HTC (KKR-CPA), and ML (NMF).

## 3. Results and discussion

**Figure 4.**
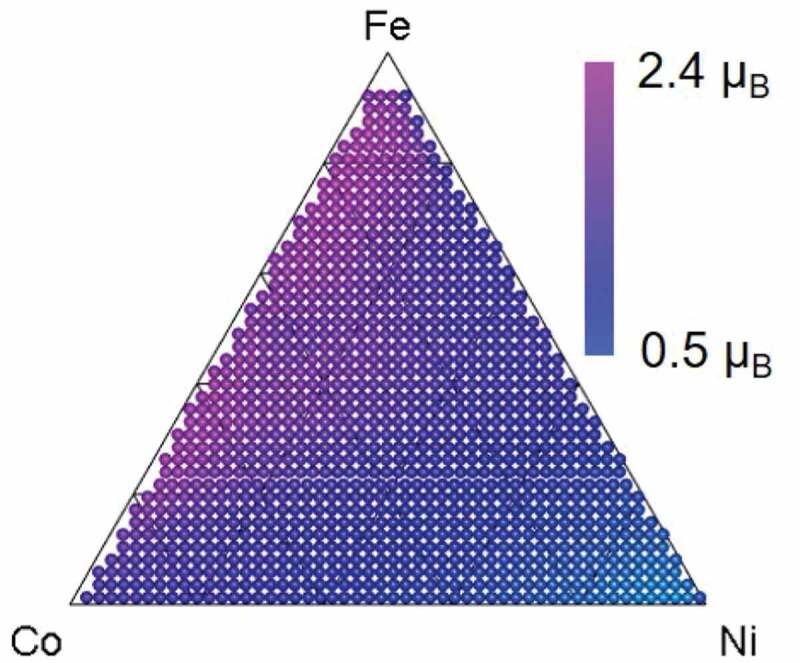
Predicted mapping with proposed method combining simple HTE (combinatorial XRD), HTC (KKR-CPA), and ML (NMF). This mapping agrees with experimental Kerr rotation mapping in Figure 1

Figure 4 shows a predicted mapping of the magnetic moment. Unlike the magnetic moment mappings of pure bcc, fcc, and hcp (Figure 2(a–c)), it agrees with the experimental mapping of Kerr rotation *θ_K_* shown in Figure 1.

Our method still has room for improvement. One approach is to reduce the other types of differences between HTE and HTC. One example is the material shape difference. The material shape for the HTE (Figure 1) was thin film while that for the HTC (Figures 2 and 4) was assumed to be bulk to reduce calculation cost. Therefore, a more accurate prediction could be obtained by assuming thin film for the HTC. Another approach is to increase the sophistication of equation (1). We simply calculated the weighted sum of structure rate *R_structure_* and magnetic moment *m_structure_* for each structural phase, so the prediction was a bit rough. By refining the equation on the basis of physics, we could obtain a more accurate prediction. If there is a sufficient amount of data for ML, we could formulize the equation by using supervised ML. Moreover, to improve convenience, we could use a calculation-based phase diagram method (e.g., CALPHAD [21]) instead of XRD and NMF, which would enable the material properties to be roughly predicted even without having a combinatorial XRD machine.

## 4. Summary

We presented a property prediction method for composition spread samples that is based on a simple HTE (combinatorial XRD), HTC (KKR-CPA), and ML (NMF). For a composition spread Fe_x_Co_y_Ni_1-x-y_ alloy sample, we demonstrated that our method enables the mapping of Kerr rotation *θ_K_* (magnetic moment *m*) to be predicted without a difficult and expensive HTE (e.g., a SMOKE experiment). This method has the potential to predict not only Kerr rotation but also other material properties and thereby shorten the development time for various materials.

## Data Availability

The data and the code that support the results within this paper and other findings of this study are available from the corresponding author upon reasonable request.
